# Disparities in Incidence of COVID-19 Among Underrepresented Racial/Ethnic Groups in Counties Identified as Hotspots During June 5–18, 2020 — 22 States, February–June 2020

**DOI:** 10.15585/mmwr.mm6933e1

**Published:** 2020-08-21

**Authors:** Jazmyn T. Moore, Jessica N. Ricaldi, Charles E. Rose, Jennifer Fuld, Monica Parise, Gloria J. Kang, Anne K. Driscoll, Tina Norris, Nana Wilson, Gabriel Rainisch, Eduardo Valverde, Vladislav Beresovsky, Christine Agnew Brune, Nadia L. Oussayef, Dale A. Rose, Laura E. Adams, Sindoos Awel, Julie Villanueva, Dana Meaney-Delman, Margaret A. Honein, COVID-19 State, Tribal, Local, and Territorial Response Team, COVID-19 State, Tribal, Local, and Territorial Response Team, Gregory Bautista, Janet Cowins, Charles Edge, Gail Grant, Robbie Gray, Sean Griffing, Nikki Hayes, Laura Hughes, Rene Lavinghouze, Sarah Leonard, Robert Montierth, Krishna Palipudi, Victoria Rayle, Andrew Ruiz, Malaika Washington, Sherri Davidson, Jennifer Dillaha, Rachel Herlihy, Carina Blackmore, Thomas Troelstrup, Laura Edison, Ebony Thomas, Caitlin Pedati, Farah Ahmed, Catherine Brown, Sarah Lyon Callo, Kathryn Como-Sabetti, Paul Byers, Victor Sutton, Zackary Moore, Sietske de Fijter, Alexia Zhang, Linda Bell, John Dunn, Stephen Pont, Keegan McCaffrey, Emily Stephens, Ryan Westergaard

**Affiliations:** 1 CDC COVID-19 Response Team.; CDC; CDC; CDC; CDC; CDC; CDC; CDC; CDC; CDC; CDC; CDC; CDC; CDC; CDC; CDC; Alabama Department of Public of Health; Arkansas Department of Health; California COVID-19 Response Team; Colorado Department of Public Health and Environment; Florida Department of Health; Florida Department of Health; Georgia Department of Public Health; Georgia Department of Public Health; Iowa Department of Public Health; Kansas Department of Health & Environment; Massachusetts Department of Public Health; Michigan Department of Health and Human Services; Minnesota Department of Health; Mississippi State Department of Health; Mississippi State Department of Health; North Carolina Department of Health and Human Services; Ohio Department of Health; Oregon Health Authority Public Health Division; South Carolina Department of Health and Environmental Control; Tennessee Department of Health; Texas Department of State Health Services; Utah Department of Health; Virginia Department of Health; Wisconsin Department of Health Services.

During January 1, 2020–August 10, 2020, an estimated 5 million cases of coronavirus disease 2019 (COVID-19) were reported in the United States.* Published state and national data indicate that persons of color might be more likely to become infected with SARS-CoV-2, the virus that causes COVID-19, experience more severe COVID-19–associated illness, including that requiring hospitalization, and have higher risk for death from COVID-19 (*1*–*5*). CDC examined county-level disparities in COVID-19 cases among underrepresented racial/ethnic groups in counties identified as hotspots, which are defined using algorithmic thresholds related to the number of new cases and the changes in incidence.^†^ Disparities were defined as difference of ≥5% between the proportion of cases and the proportion of the population or a ratio ≥1.5 for the proportion of cases to the proportion of the population for underrepresented racial/ethnic groups in each county. During June 5–18, 205 counties in 33 states were identified as hotspots; among these counties, race was reported for ≥50% of cumulative cases in 79 (38.5%) counties in 22 states; 96.2% of these counties had disparities in COVID-19 cases in one or more underrepresented racial/ethnic groups. Hispanic/Latino (Hispanic) persons were the largest group by population size (3.5 million persons) living in hotspot counties where a disproportionate number of cases among that group was identified, followed by black/African American (black) persons (2 million), American Indian/Alaska Native (AI/AN) persons (61,000), Asian persons (36,000), and Native Hawaiian/other Pacific Islander (NHPI) persons (31,000). Examining county-level data disaggregated by race/ethnicity can help identify health disparities in COVID-19 cases and inform strategies for preventing and slowing SARS-CoV-2 transmission. More complete race/ethnicity data are needed to fully inform public health decision-making. Addressing the pandemic’s disproportionate incidence of COVID-19 in communities of color can reduce the community-wide impact of COVID-19 and improve health outcomes.

This analysis used cumulative county-level data during February–June 2020, reported to CDC by jurisdictions or extracted from state and county websites and disaggregated by race/ethnicity. Case counts, which included both probable and laboratory-confirmed cases, were cross-referenced with counts from the HHS Protect database (https://protect-public.hhs.gov/). Counties missing race data for more than half of reported cases (126) were excluded from the analysis.^§^ The proportion of the population for each county by race/ethnicity was calculated using data obtained from CDC WONDER (*6*). For each underrepresented racial/ethnic group, disparities were defined as a difference of ≥5% between the proportion of cases and the proportion of the population consisting of that group or a ratio of ≥1.5 for the proportion of cases to the proportion of the population in that racial/ethnic group. The county-level differences and ratios between proportion of cases and the proportion of population were used as a base for a simulation accounting for missing data using different assumptions of racial/ethnic distribution of cases with unknown race/ethnicity. An intercept-only logistic regression model was estimated for each race/ethnicity category and county to obtain the intercept regression coefficient and standard error. The simulation used the logistic regression-estimated coefficient and standard error to produce an estimated mean and confidence interval (CI) for the percentage difference between and ratio of proportions of cases and population. This simulation was done for each racial/ethnic group within each county. The lower bound of the CI was used to identify counties with disparities (as defined by percentage differences or ratio). The mean of the estimated differences and mean of the estimated ratios were calculated for all counties with disparities. Analyses were conducted using SAS software (version 9.4; SAS Institute).

During June 5–18, a total of 205 counties in 33 states were identified as hotspots. These counties have a combined total population of 93.5 million persons, and approximately 535,000 cumulative probable and confirmed COVID-19 cases. Among the 205 identified hotspot counties, 79 (38.5%) counties in 22 states, with a combined population of 27.5 million persons and approximately 162,000 COVID-19 cases, had race data available for ≥50% of cumulative cases and were included in the analysis (range = 51.3%–97.4%). Disparities in cases were identified among underrepresented racial/ethnic groups in 76 (96.2%) analyzed counties (Table 1). Disparities among Hispanic populations were identified in approximately three quarters of hotspot counties (59 of 79, 74.7%) with approximately 3.5 million Hispanic residents (Table 2). Approximately 2.0 million black persons reside in 22 (27.8%) hotspot counties where black residents were disproportionately affected by COVID-19, approximately 61,000 AI/AN persons live in three (3.8%) hotspot counties where AI/AN residents were disproportionately affected by COVID-19, nearly 36,000 Asian persons live in four (5.1%) hotspot counties where Asian residents were disproportionately affected by COVID-19, and approximately 31,000 NHPI persons live in 19 (24.1%) hotspot counties where NHPI populations were disproportionately affected by COVID-19.

**TABLE 1:** Total population and racial/ethnic disparities* in cumulative COVID-19 cases among 79 counties identified as hotspots during June 5–18, 2020, with any disparity identified — 22 states, February–June 2020

State	No. of persons living in analyzed hotspot counties*	No. of (col %) hotspot counties analyzed^†^	No. of counties with disparities in COVID-19 cases among each racial/ethnic group^§^				
			Hispanic	Black	NHPI	Asian	AI/AN
Alabama	500,000–1,000,000	1 (1.3)	—	1	—	—	—
Arizona	1,000,000–3,000,000	5 (6.3)	3	—	—	—	3
Arkansas	500,000–1,000,000	4 (5.1)	4	—	2	—	—
California	1,000,000–3,000,000	1 (1.3)	1	—	—	—	—
Colorado	100,000–500,000	1 (1.3)	1	—	1	—	—
Florida	>3,000,000	6 (7.6)	3	2	—	—	—
Georgia	100,000–500,000	1 (1.3)	1	—	—	—	—
Iowa	50,000–100,000	1 (1.3)	1	—	—	—	—
Kansas	500,000–1,000,000	2 (2.5)	2	—	2	—	—
Massachusetts	500,000–1,000,000	2 (2.5)	—	2	—	—	—
Michigan	1,000,000–3,000,000	5 (6.3)	—	5	1	—	—
Minnesota	<50,000	1 (1.3)	1	1	1	1	—
Mississippi	100,000–500,000	2 (2.5)	1	2	—	—	—
North Carolina	>3,000,000	18 (22.8)	18	—	3	1	—
Ohio	1,000,000–3,000,000	3 (3.8)	3	2	—	1	—
Oregon	1,000,000–3,000,000	6 (7.6)	6	1	4	1	—
South Carolina	1,000,000–3,000,000	9 (11.4)	6	4	2	—	—
Tennessee	500,000–1,000,000	3 (3.8)	3	—	—	—	—
Texas	500,000–1,000,000	2 (2.5)	—	1	—	—	—
Utah	1,000,000–3,000,000	4 (5.1)	4	1	3	—	—
Virginia	<50,000	1 (1.3)	—	—	—	—	—
Wisconsin	100,000–500,000	1 (1.3)	1	—	—	—	—
**Total (approximate)**	**27,500,000**	**79 (100)**	**59**	**22**	**19**	**4**	**3**

**Abbreviations:** AI/AN = American Indian/Alaska Native; COVID-19 = coronavirus disease 2019; NHPI = Native Hawaiian/other Pacific Islanders.

* Disparities were defined as percentage difference of ≥5% between the proportion of cases and the proportion of the population or a ratio ≥1.5 for the proportion of cases to the proportion of the population) for underrepresented racial/ethnic groups in each county.

^†^ Counties with race/ethnicity data available for ≥50% of cases.

^§^ Racial/ethnic groups are not mutually exclusive in a given county.

**TABLE 2:** Number of persons in each racial/ethnic group living in 79 counties identified as hotspots during June 5–18, 2020 with disparities* — 22 states, February–June 2020

Racial/Ethnic group	No. (%)^†^ of counties with disparities^§^ identified	Approximate no. of persons living in hotspot counties with disparities
Hispanic/Latino	59 (74.7)	3,500,000
Black/African American	22 (27.8)	2,000,000
American Indian/Alaska Native	3 (3.8)	61,000
Asian	4 (5.1)	36,000
Native Hawaiian/Other Pacific Islander	19 (24.1)	31,000
**Total**	**—**	**5,628,000**

**Abbreviation:** COVID-19 = coronavirus disease 2019.

* Disparities were defined as percentage difference of ≥5% between the proportion of cases and the proportion of the population or a ratio ≥1.5 for the proportion of cases to the proportion of the population) for underrepresented racial/ethnic groups in each county.

**^†^** Percentage of the 79 counties.

^§^ Disparities are in respective racial/ethnic groups and are not mutually exclusive; some counties had disparities in more than one racial/ethnic group.

The mean of the estimated differences between the proportion of cases and proportion of the population consisting of each underrepresented racial/ethnic group in all counties with disparities ranged from 4.5% (NHPI) to 39.3% (AI/AN) (Table 3). The mean of the estimated ratio of the proportion of cases to the proportions of population were also generated for each underrepresented racial/ethnic group and ranged from 2.3 (black) to 8.5 (NHPI).

**TABLE 3:** Proportion of cumulative COVID-19 cases compared with proportion of population in 79 counties identified as hotspots during June 5–18, 2020 with racial/ethnic disparities* — 22 states February–June 2020

Racial/Ethnic group	Mean of estimated differences, ^†^ % (range)	Mean of estimated ratios of proportion of cases to proportion of population^§^ (range)
Hispanic/Latino	30.2 (8.0–68.2)	4.4 (1.2–14.6)
Black/African American	14.5 (2.3–31.7)	2.3 (1.2–7.0)
American Indian/Alaska Native	39.3 (16.4–57.9)	4.2 (1.9–6.4)
Asian	4.7 (2.7–6.8)	2.9 (2.0–4.7)
Native Hawaiian/Other Pacific Islander	4.5 (0.1–31.5)	8.5 (2.7–18.4)

**Abbreviation:** COVID-19 = coronavirus disease 2019. * Disparities were defined as percentage difference of ≥5% between the proportion of cases and the proportion of the population or a ratio ≥1.5 for the proportion of cases to the proportion of the population) for underrepresented racial/ethnic groups in each county.

^†^ The mean of the estimated differences between the proportion of cases in a given racial/ethnic group and the proportion of persons in that racial/ethnic group in the overall population among all counties with disparities identified by the analysis. For example, if Hispanic/Latino persons make up 20% of the population in a given county and 30% of the cases in that county, then the difference would be 10% and the county is considered to have a disparity.

^§^ The ratio of the estimated proportion of cases to the proportion of population for each racial/ethnic group among all counties with disparities identified by the analysis. For example, if American Indian/Alaskan Native persons made up 0.5% of the population in a given county and 1.5% of the cases in that county, then the ratio of proportions would be 3.0, and the county is considered to have a disparity.

## Discussion

These findings illustrate the disproportionate incidence of COVID-19 among communities of color, as has been shown by other studies, and suggest that a high percentage of cases in hotspot counties are among persons of color (*1*–*5*,*7*). Among all underrepresented racial/ethnic groups in these hotspot counties, Hispanic persons were the largest group living in hotspot counties with a disparity in cases identified within that population (3.5 million persons). This finding is consistent with other evidence highlighting the disproportionate incidence of COVID-19 among the Hispanic population (*2*,*7*). The disproportionate incidence of COVID-19 among black populations is well documented (*1*–*3*). The findings from this analysis align with other data indicating that black persons are overrepresented among COVID-19 cases, associated hospitalizations, and deaths in the United States. The analysis found few counties with disparities among AI/AN populations. This finding is likely attributable to the smaller proportions of cases and populations of AI/AN identified in hotspot counties, as well as challenges with data for this group, including a lack of surveillance data and misclassification problems in large data sets.^¶^ Asian populations were disproportionately affected by COVID-19 in a small number of hotspot counties. Few studies have assessed COVID-19 disparities among Asian populations in the United States.** The Asian racial category is broad, and further subgroup analyses might provide additional insights regarding the incidence of COVID-19 in this population. Disparities in COVID-19 cases in NHPI populations were identified in nearly one quarter of hotspot counties. For some hotspot counties with small NHPI populations, this finding might be related, in part, to the analytic methodology used. Using a ratio of ≥1.5 in the proportion of population and proportion of cases to indicate disparities is sensitive to small differences in these groups. More complete county-level race/ethnicity data are needed to fully evaluate the disproportionate incidence of COVID-19 among communities of color.

Disparities in COVID-19–associated mortality in hotspot counties were not assessed because the available county-level mortality data disaggregated by race/ethnicity were not sufficient to generate reliable estimates. Existing national analyses highlight disparities in mortality associated with COVID-19; similar patterns are likely to exist at the county level (*5*). As more complete data are made available in the future, county-level analyses examining disparities in mortality might be possible. COVID-19 disparities among underrepresented racial/ethnic groups likely result from a multitude of conditions that lead to increased risk for exposure to SARS-CoV-2, including structural factors, such as economic and housing policies and the built environment,^††^ and social factors such as essential worker employment status requiring in-person work (e.g., meatpacking, agriculture, service, and health care), residence in multigenerational and multifamily households, and overrepresentation in congregate living environments with an increased risk for transmission (*4*,*7*–*9*). Further, long-standing discrimination and social inequities might contribute to factors that increase risk for severe disease and death, such as limited access to health care, underlying medical conditions, and higher levels of exposure to pollution and environmental hazards^§§^ (*4*). The conditions contributing to disparities likely vary widely within and among groups, depending on location and other contextual factors.

Rates of SARS-CoV-2 transmission vary by region and time, resulting in nonuniform disease outbreak patterns across the United States. Therefore, using epidemiologic indicators to identify hotspot counties currently affected by SARS-CoV-2 transmission can inform a data-driven emergency response. Tailoring strategies to control SARS-CoV-2 transmission could reduce the overall incidence of COVID-19 in communities. Using these data to identify disproportionately affected groups at the county level can guide the allocation of resources, development of culturally and linguistically tailored prevention activities, and implementation of focused testing efforts.

The findings in this report are subject to at least five limitations. First, more than half of the hotspot counties did not report sufficient race data and were therefore excluded from the analysis. In addition, many hotspot counties included in the analyses were missing data on race for a significant proportion of cases (mean = 28.3%; range = 2.6%–48.7%). These data gaps might result from jurisdictions having to reconcile data from multiple sources for a large volume of cases while data collection and management processes are rapidly evolving.^¶¶^ Second, health departments differ in the way race/ethnicity are reported, making comparisons across counties and states more difficult. Third, differences in how race/ethnicity data are collected (e.g., self-report versus observation) likely varies by setting and could lead to miscategorization. Fourth, differences in access to COVID-19 testing could lead to underestimates of prevalence in some underrepresented racial/ethnic populations. Finally, the number of cases that had available race/ethnicity data for the period of study of hotspots (June 5–18) was too small to generate reliable estimates, so cumulative case counts by county during February–June 2020 were used to identify disparities. This approach describes the racial/ethnic breakdown of cumulative cases only. Therefore, these data might not provide an accurate estimate of disparities during June 5–18, which could be under- or overestimated, or change over time.

Developing culturally responsive, targeted interventions in partnership with trusted leaders and community-based organizations within communities of color might reduce disparities in COVID-19 incidence. Increasing the proportion of cases for which race/ethnicity data are collected and reported can help inform efforts in the short-term to better understand patterns of incidence and mortality. Existing health inequities amplified by COVID-19 highlight the need for continued investment in communities of color to address social determinants of health*** and structural racism that affect health beyond this pandemic (*4*,*8*). Long-term efforts should focus on addressing societal factors that contribute to broader health disparities across communities of color.

**Summary**

**What is already known about this topic?**

Long-standing health and social inequities have resulted in increased risk for infection, severe illness, and death from COVID-19 among communities of color.

**What is added by this report?**

Among 79 counties identified as hotspots during June 5–18, 2020 that also had sufficient data on race, a disproportionate number of COVID-19 cases among underrepresented racial/ethnic groups occurred in almost all areas during February–June 2020.

**What are the implications for public health practice?**

Identifying health disparities in COVID-19 hotspot counties can inform testing and prevention efforts. Addressing the pandemic’s disproportionate incidence among communities of color can improve community-wide health outcomes related to COVID-19.

## Footnotes

* https://www.cdc.gov/coronavirus/2019-ncov/cases-in-us.html.

† Hotspot counties are defined as those meeting all of the following baseline criteria: 1) >100 new COVID-19 cases in the most recent 7 days, 2) an increase in the most recent 7-day COVID-19 incidence over the preceding 7-day incidence, 3) a decrease of <60% or an increase in the most recent 3-day COVID-19 incidence over the preceding 3-day incidence, and 4) the ratio of 7-day incidence to 30-day incidence exceeds 0.31. In addition, hotspots must have met at least one of the following criteria: 1) >60% change in the most recent 3-day COVID-19 incidence, or 2) >60% change in the most recent 7-day incidence.

§ Data from 10 of the 126 excluded counties were excluded due to pending data questions.

¶ https://aspe.hhs.gov/execsum/gaps-and-strategies-improving-american-indianalaska-nativenative-american-data.

** https://www.healthaffairs.org/do/10.1377/hblog20200708.894552/full/.

†† The built environment includes the physical makeup of where persons live, learn, work, and play, including homes, schools, businesses, streets and sidewalks, open spaces, and transportation options. The built environment can influence overall community health and individual. Behaviors, such as physical activity and healthy eating. https://www.cdc.gov/nccdphp/dnpao/state-local-programs/built-environment-assessment/.

§§ https://www.medrxiv.org/content/10.1101/2020.04.05.20054502v2.

¶¶ https://www.hhs.gov/about/news/2020/06/04/hhs-announces-new-laboratory-data-reporting-guidance-for-covid-19-testing.html.

*** https://www.healthypeople.gov/2020/topics-objectives/topic/social-determinants-of-health.
